# The Development of a Psychometrically Valid and Reliable Questionnaire to Assess Nutrition Knowledge Related to Pre-Schoolers

**DOI:** 10.3390/nu12071964

**Published:** 2020-07-01

**Authors:** Jeanette Rapson, Cathryn Conlon, Kathryn Beck, Pamela von Hurst, Ajmol Ali

**Affiliations:** School of Sport, Exercise and Nutrition, Massey University, Auckland 0632, New Zealand; J.Rapson@massey.ac.nz (J.R.); C.Conlon@massey.ac.nz (C.C.); K.L.Beck@massey.ac.nz (K.B.); P.R.vonHurst@massey.ac.nz (P.v.H.)

**Keywords:** childhood obesity, childcare, health knowledge, attitude, practice, surveys and questionnaires

## Abstract

With rising childcare enrollments, caregivers have a unique opportunity to promote children’s nutrition education and healthy eating. Accurately identifying nutrition knowledge gaps amongst caregivers is necessary for professional development planning. Our aim was to design an early childhood education and care (ECEC) teacher nutrition knowledge questionnaire that satisfies psychometric criteria of validity and reliability. Items were based on the New Zealand Ministry of Health dietary guidelines, literature and expert advice. University students in their final year of a Bachelor of Science (BSc) in Human Nutrition (*n* = 40), and students with no nutrition background (*n* = 51) completed the questionnaire to assess construct validity; 35 BSc nutrition students completed the questionnaire two weeks later to assess reliability. The Mann-Whitney-U test and a median-split table assessed construct validity; Pearson’s product-moment correlation assessed test-retest reliability. Nutrition students achieved higher total and subcategory scores (*p* < 0.01). All nutrition students scored above the median of the combined group; 82% of non-nutrition students scored below the median. In testing reliability, first and second administration median scores for total and subcategories were significantly correlated (*r* = 0.43–0.78; *p* < 0.01). The questionnaire achieved construct validity and test-retest reliability and measured ECEC teachers’ nutrition knowledge for preschoolers.

## 1. Introduction

Participation in early childhood education and care (ECEC) continues to rise across globally [1]. In New Zealand, the number of children attending education and care services increased to 135,237 in 2019 (up 0.4% from 2018), with an average attendance of 23.3 h per week [2]. Therefore, these environments are increasingly considered to impact a child’s dietary and physical activity patterns [3,4]. The childcare nutrition environment, however, is often suboptimal with evidence of low adherence to recommended nutrition-related behaviours and childcare menus not meeting nutrition guidelines for quantity, variety, and limiting ‘sometimes’ and ‘occasional’ foods [4,5,6,7]. Providing guidance to caregivers and ECEC teachers on appropriate nutrition and physical activity for preschoolers is recommended to ensure children establish good nutrition and physical activity behaviours that maximise child development and reduce the risk of developing obesity [8]. A first step for providing this support is to accurately identify knowledge deficits, in which using a valid and reliable ECEC teacher nutrition and physical activity knowledge questionnaire is useful. The current range of ECEC teacher nutrition and physical activity knowledge questionnaires that demonstrate at least construct and content validity are relatively out-of-date (ranging from 1972–2010) [9,10,11,12], mostly related to US teachers [9,10,11,12,13,14,15,16,17,18,19,20], and/or often include content that is not specific to pre-schoolers’ nutrition nor physical activity [11,17,18,21]. Other knowledge questionnaires focusing on the diet of young children have been used to assess parental knowledge, but these are limited in scope, lack validity, and feature items that are overly specialised, for example, asking parents to decide if the following statement is correct: “If one should pay attention to the weight of a pre-schooler, it is preferable to substitute potatoes by rice and pasta” [22,23]. Through a semi-structured literature review of caregivers’ nutrition knowledge, we identified only four [9,10,12,24] of 15 studies used nutrition/physical activity knowledge questionnaires that showed content and construct validity; four were partially validated (no construct validity) [16,19,20,25], and the remaining seven [13,14,15,17,18,21,26] either did not describe validation methods or did not appear to be validated. These characteristics may have limited the quality of evidence for ECEC teachers’ nutrition knowledge.

Psychometrics, the science of maximising quality assessment, provides criteria for validating a knowledge questionnaire [27]. Common forms of validity used for nutrition knowledge questionnaires include content and construct validity and test-retest reliability [9,10,11,16,28,29]. Content validity refers to how well the questionnaire content matches with the questionnaire purpose [27,30], and is usually assessed by using feedback from experts in the instrument’s relevant field. Construct validity refers to the extent that the measure’s variance relates with the variance of its underlying construct or idea of an attribute [30]. This means that a group of experts of the attribute in question (e.g., nutritionists) should score significantly higher than a group of non-experts (e.g., non-nutritionists) completing the same questionnaire [29]. Test-retest reliability ensures scores do not significantly change when completed by the same sample on two separate occasions [27], and is typically assessed by the Pearson’s product-moment correlation method [27,28,29].

In New Zealand, no studies prior to this investigation have objectively measured ECEC teachers’ nutrition knowledge for preschoolers, which may partly be due to a lack of suitable measuring tools. To help address this issue, the availability of a psychometrically valid and reliable questionnaire that can measure ECEC teachers’ nutrition knowledge would be useful. Therefore, the aim of this study is to design an ECEC teachers’ nutrition knowledge questionnaire that satisfies psychometric criteria of content and construct validity and reliability.

## 2. Materials and Methods 

### 2.1. Study Design and Participants

The protocol for this validation study followed psychometric guidelines for constructing questionnaires [27,30] and methods of previous studies [28,29]. To assure content validity, the questionnaire was developed alongside an expert panel of three nutrition and exercise science experts and three key ECEC stakeholders (i.e., childcare managers/head teachers) in New Zealand. Following semi-structed interview questions and using a feedback form, items were discussed in terms of relevancy, difficultly, readability, accuracy, and scope; panel consensus confirmed the final questionnaire. 

To measure construct validity, a class of nutrition university students and those identified through a screening questionnaire as non-nutrition university students were invited to participate in the study. Nutrition students who agreed, completed the questionnaire online in a controlled setting with the primary author present. In addition, at the end of the testing session, the nutrition students provided general verbal feedback on the questionnaire format and readability, which was recorded by the researcher for further content validity testing. Non-nutrition university students were invited through other faculties within the university (e.g., business school) and social media to complete the questionnaire once via e-mail; their responses were compared to the nutrition students’ first occasion responses. 

To test reliability, the nutrition students completed the same questionnaire two weeks later. This time period is considered long enough to prevent participants remembering their previous responses, yet short enough to avoid real change in nutrition knowledge [28,29]. As test-retest reliability in its simplest form requires two data sets from different time points from the same group [27], only the nutrition students completed the second administration. Principles of Classical Test Theory were followed, including a basic assumption that the observed score of a person on a test is composed of a true score and an error, and so allows for reliability testing [31]. The Classical Test Theory model fits the questionnaire design, which adds together the scores of individual items to reach a total score [32]. As per previous validation studies [28,29], the sample sizes were reflective of typical classroom enrolments. Ethical approval was provided by the Massey University Human Ethics Committee: Northern (application No. 15/36).

### 2.2. Questionnaire Design

The questionnaire design was based on a literature search of existing ECEC nutrition knowledge questionnaires, nutrition guidelines for preschoolers [33,34], input from the ECEC stakeholders, and nutrition and exercise science experts. Following consultation with the expert panel, four nutrition knowledge domains for testing were identified, including: the recommended servings per food group; suitable food and beverage choices; appropriate portion versus serving size; and awareness of current child nutrition guidelines/resources. Experts agreed that all items should be specific to nutrition for preschoolers, rather than general nutrition knowledge for adults. Rust and Golombok’s [27] knowledge questionnaire guidelines were followed, for example, providing clear instructions as to how to choose a response (i.e., “please select one”). Nutrition-related perspective items (e.g., “meal times should be fun”) were included to improve questionnaire design and align with previous questionnaires [9,11,12,14,16,18,21,26]. These items were not assessed as they focused on perspectives rather than knowledge but were reviewed for content validity by the expert panel. Demographic items included gender, age, ethnicity, previous training in nutrition, highest qualification, and current course of study. 

There were approximately 20 revisions of the questionnaire, with the first version featuring 56 items and the Healthy Food Guide’s serving chart [35], while the final version had eliminated 16 items and used the Ministry of Health’s serving examples [34] to better align with the Ministry of Health’s guideline documents. The final questionnaire was placed online using Qualtrics survey software. Skip-logic questions, by which a set of conditions triggered skipping ahead in the survey, were used to reduce respondent burden, thus improve response rates. For example, if a participant selected that they were not aware of the New Zealand nutrition guidelines for preschoolers, then the next question asking if they had used the guidelines was skipped. Previously, ECEC staff have indicated that a survey length of 20 min was a reason not to participate in the study [36]. Since it took the nutrition students approximately 25 min to complete the questionnaire, five nutrition knowledge items that appeared cumbersome (required reading a case study) were omitted to reduce completion time. The nutrition students’ feedback after completing the questionnaire resulted in no other changes. The final 40-item questionnaire took approximately 15 min to complete. Nineteen items objectively measured nutrition and were tested for validity; and were organised into subcategories: servings (6 questions; maximum score 6), food choices (7 questions; maximum score 25), portions (5 questions; maximum score 5), and resources (1 question; maximum score 1). Participants could score a maximum total of 37.

**Figure 1:**
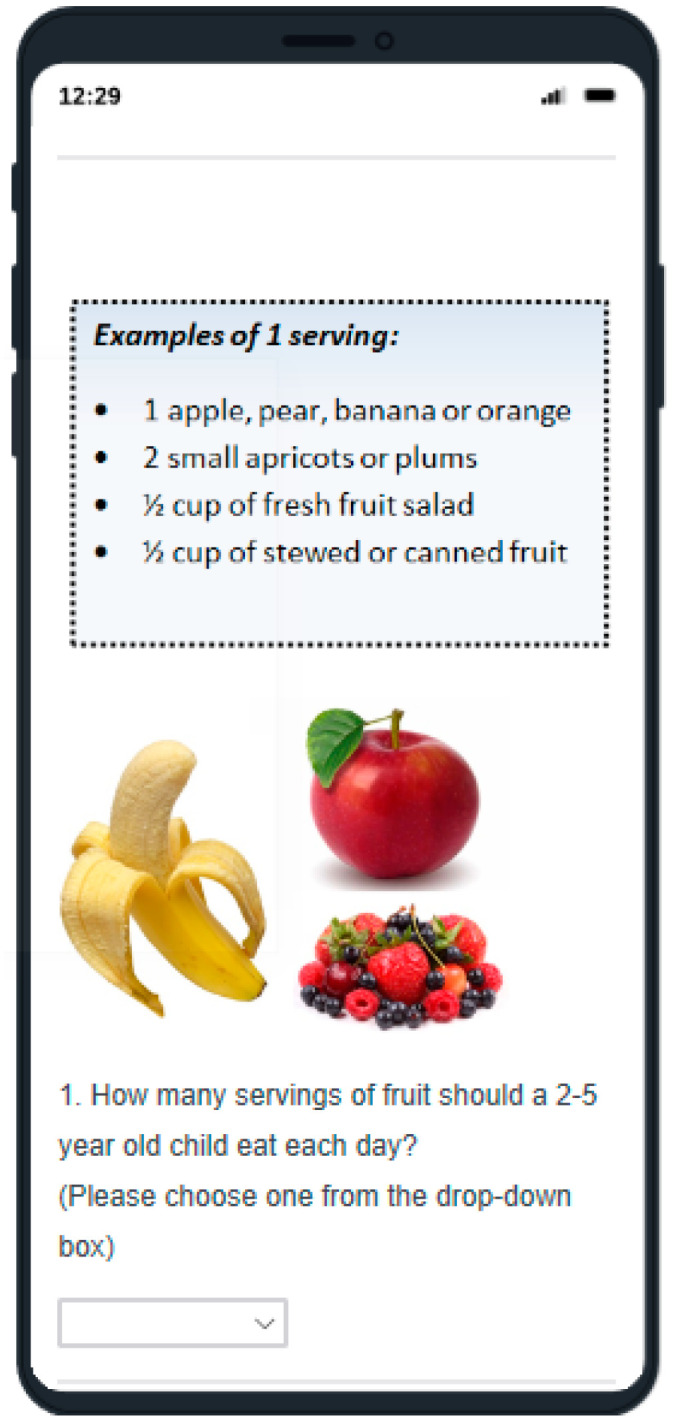
Example question using a mobile phone view.

To ensure the questionnaire was at an appropriate level, testing concepts that seemed historically difficult to grasp were avoided, such as recommended daily intakes (RDI) [16,18,37]. Moreover, RDI may be phasing out from nutrition resources [34,38,39], so appeared less relevant. Examples of serving sizes were taken from nutrition guidelines [33,34] to standardise answers for the servings’ subcategory. Due to the increasing use of digital technologies in education [40], the questionnaire was in an online digital and visually appealing format that could be completed using a computer or smart mobile devices (e.g., tablets, smartphones) (Figure 1). Images were royalty-free or created by the primary author. Lunchbox food images were based on previous research [41]. To maximise data quality and increase response rates, the questionnaire favoured closed-questions (quantitative data); however, open-questions (qualitative) were included (e.g., “please specify”) to clarify answers [42]. However, for the knowledge items, qualitative data was only collected when asking if there were any other suitable drinks or snacks that are appropriate for preschoolers. Forced responses and request responses were used to minimise missing data; however, an option “choose not to answer” was provided. Items were coded numerically, with nutrition knowledge items being given a “+1” score for a correct answer and “0” for an incorrect, “choose not to answer” or “not sure” response. For the purpose of scoring, a qualitative response that was contradictory to nutrition guidelines was also scored as “0” for incorrect, while a response in alignment was scored “+1”. The sum of these answers provided total and subcategory knowledge scores. A copy of the questionnaire is available (see Supplementary File S1).

### 2.3. Statistical Analysis

Statistical analysis was conducted using IBM SPSS (version 24.0 IBM Corp., Armonk, NY, USA, 2016). Descriptive statistics summarised participant characteristics and nutrition knowledge scores. As histograms and Kolmogorov-Smirnov tests showed that data were not normally distributed and no improvements were seen with data transformation, the Mann-Whitney-U test was used to test construct validity and median (25th, 75th percentiles) scores were reported. Effect size was calculated using Rosenthal’s [43] formula and interpreted using Cohen’s [44] guidelines on effect size *r* (small: 0.1, medium: 0.3, large: 0.5). A median-split table was used as a second measure of construct validity. Pearson’s product-moment correlation coefficient assessed test-retest reliability (nutrition students’ data only) as this is a straight-forward method previously used [28,29] and recommended by Rust and Golombok [27]. Intraclass correlations were not conducted because when raters/assessors are adequately informed and trained to make appropriate assessments (as in this study), a Pearson’s product-moment correlation and intraclass correlation should produce similar results [45]. Correlations (*r*) were interpreted using Cohen’s [44] guidelines for interpreting *r* (small: 0.1, medium: 0.3, large: 0.5). The significance level was *p* < 0.05 for all tests. 

## 3. Results

### 3.1. Participants

The total sample comprised 77 females and 13 males; 58% were 16–24 years and 67% were of New Zealand European ethnicity. The nutrition students (*n* = 40) included 35 females; 68% were 16–24 years and 63% were New Zealand European. The non-nutrition students (*n* = 51) included 42 females; 51% were 16–24 years and 71% were New Zealand European. 

### 3.2. Content Validity

Content validity was considered satisfactory due to the organised design methods involving experts [29]. Written and verbal feedback collected from the panel of experts/stakeholders and the 40 nutrition students confirmed content validity.

### 3.3. Construct Validity

Nutrition students’ median scores for total and subcategory nutrition knowledge was higher than the non-nutrition students (*p* < 0.01; Table 1). The median-split table (Table 2) shows 100% of nutrition students scored better than the median of the combined group, while 72% of non-nutrition students scored below the median.

### 3.4. Test-Retest Reliability

First and second median scores for overall and subcategory scores (except the resource subcategory) were significantly correlated (*p* < 0.01); correlation coefficients ranged from *r* = 0.43–0.78. There was a small, but non-significant correlation (*r* = 0.12, *p* = 0.503) for the resources subcategory (Table 3). 

## 4. Discussion

This study aimed to develop a psychometrically valid and reliable questionnaire to assess ECEC teacher nutrition knowledge. As the nutrition students scored significantly higher overall and for each subcategory, the questionnaire demonstrated satisfactory construct validity. Furthermore, 100% of nutrition students scored above the median of the combined group, while 72% of non-nutrition students scored below the median. The questionnaire also met satisfactory content validity; researchers may consider the organised methods used (including a literature review and expert advice) to indicate “undoubtedly high” [29] content validity; however, we remain conservative in view of no other benchmarks available. The test-retest reliability of this tool proved acceptable levels for reliability, with all correlations of medium to large strength, except for the ‘resources’ subcategory. 

The construct validity reported here suggests that this tool adequately measures what it is supposed to measure, which is ECEC teacher’s nutrition knowledge for preschoolers. It is difficult to compare results to those of previous studies given the dearth of validated ECEC teacher nutrition knowledge questionnaires, heterogeneity between study designs, and limited detail provided in reporting. Nevertheless, these findings may be comparable to early questionnaires [9,10], for example, Gillis and Sabry [10] found 44 students who had completed at least one course in nutrition obtained a significantly higher (*p* < 0.005) total average knowledge score of 15.9 ± 2.7 on the study’s 20-item childcare teacher nutrition knowledge questionnaire, compared with 29 students who had not studied nutrition and scored 11.0 ± 2.9 [10]. In our study, it was unclear as to why nine non-nutrition students scored above the median as demographic data was similar between groups and all indicated that they had not attained any previous training in nutrition or physical activity for young children. Further investigation of non-nutrition students’ specific area of expertise may have provided more insight, but with the majority scoring below the median, results still indicated the expected knowledge difference between the two groups.

Our test-retest reliability results suggest that one could trust the tool to accurately measure nutrition knowledge when administered to caregivers in the future. These results may be comparable to those of Dias et al. [46], which seems to be the only other study to have reported on test-retest reliability of a questionnaire measuring caregivers’ nutrition knowledge for preschoolers. In their validation analysis, a paired t-test found that all knowledge scores were higher in the questionnaire’s retest administration, but differences were not significant for nursery staff (*p* = 0.05), and the intraclass correlation coefficient (ICC) was 0.76, which is just above the acceptable level of 0.7 [46]. As a sample size of fifty participants was needed for estimating an ICC of 0.8 with a 95% CI ± 0.1 for two repeated measures, their small sample of 21 nursery staff may have limited results. In our study, the small and non-significant correlation for the ‘resources’ subcategory suggested that these items were less reliable; however, they were retained in the interest of content validity, which is not an uncommon practice if overall reliability is unaffected [29]. Furthermore, scores for this section might be less indicative of ECEC teachers’ nutrition knowledge for preschoolers, as it only consisted of one item asking if New Zealand had specific nutrition and physical activity guidelines, rather than testing the content of these guidelines. 

A strength of this study is that it is the first to develop a psychometrically valid nutrition knowledge questionnaire that can be used amongst New Zealand ECEC teachers. The methodology is detailed regarding questionnaire design, which should be useful to those wanting to use the questionnaire or create a similar tool. As university students tend to share similar characteristics (e.g., age, geographic location), yet differ in fields of expertise, they were an ideal population group to test validity. The nutrition students were in their third and final year of university study, therefore, could be considered experts compared to the group with no nutrition training (non-experts). In addition, as the benchmark qualification for New Zealand qualified ECEC teachers is at tertiary level [47], using tertiary education students in a university setting was considered comparable to the target population. Findings may be limited by the different testing conditions for each group (e.g., the nutrition students completed the questionnaires in a classroom scenario, whereas the non-nutrition students were participating remotely). However, with similar completion times and clear instructions to answer items without reference material, it is likely that differences in setting did not impact results.

The Classical Test Theory has traditionally been used to assess the validity of nutrition knowledge questionnaires. It is also based on the assumption that all items equally measure an individual’s nutrition knowledge and is unable to differentiate individuals with the same ‘total’ score. Such bias may have been addressed with Rasch analysis, which has recently been used to develop a brief nutrition knowledge questionnaire for athletes [48,49]. In situations where respondents receive the same ‘total’ score, Rasch analysis can take into account differences in knowledge levels according to which questions were correctly answered or question difficulty [48,49,50,51]. While including this analysis may have strengthened results and given more detailed diagnostics of how to improve the questionnaire, authors recommend it be carried out in a larger sample size (e.g., 240 participants) [49,50].

This questionnaire covers current New Zealand nutrition recommendations for preschoolers, thus represents a comprehensive and objective assessment of knowledge in this area. Previous ECEC nutrition knowledge questionnaires were relatively out-of-date, poorly validated, and/or non-specific for preschoolers’ nutrition, with none available for use in New Zealand. Given the validity and currency of this tool, it may be adapted for use in other countries, especially those with similar nutrition guidelines (e.g., UK, Australia). This questionnaire may serve as a template for researchers and authorities investigating parents’, caregivers, practitioners’, and/or teachers’ nutrition knowledge across other settings, including primary and secondary schools. For example, a preschool-based intervention in the Netherlands, which recognises the important role that ECEC teachers play in promoting children’s healthy eating and physical activity, is currently underway [52]. An assessment of improved teacher nutrition knowledge is one of the key indicators of the intervention’s success, and so the results of this study could be useful to inform their assessment methods. Meanwhile, if a similar intervention were to be conducted in New Zealand, the tool can now be used, and the comparability of studies may be possible. Given that ECEC teachers work in a busy environment and may have limited resources [4], we would recommend that ECEC teachers are given support to identify an appropriate time and place to complete this questionnaire. It is important to stress to teachers that the tool is designed to inform future training and professional development, rather than to degrade or embarrass them. As the questionnaire is written in English, additional support when interpreting the questions using a translator may be required where English is not a first language. Finally, the potential impact of this tool for the New Zealand and global ECEC sector may largely be for the development of teacher qualification programmes. When used to accurately identify knowledge gaps amongst ECEC teachers (both practicing or in training) this tool could: (1) help justify a greater amount of time allocated to teacher nutrition training; (2) develop specific learning objectives and content for teacher course curriculum, and: (3) be a valuable assessment tool for ECEC teacher competency testing.

## 5. Conclusions

This study is the first to develop a psychometrically valid and reliable ECEC teachers’ nutrition knowledge for preschoolers questionnaire for use in New Zealand. It is intended that this tool be used by researchers, practitioners or ECEC providers aiming to investigate ECEC teachers’ nutrition knowledge and related perspectives; as we have demonstrated elsewhere [53]. Identifying knowledge gaps is a first step for providing ECEC teachers with the relevant knowledge to support and implement children’s healthy eating practices whilst in childcare. This may be one of many important strategies for obesity prevention in early life. 

## Figures and Tables

**Table 1:** Mann-Whitney-U test comparing nutrition knowledge scores between nutrition and non-nutrition students.

	Nutrition Students (*n* = 40) ^1^	Non-nutrition Students (*n* = 51) ^1^	*U*	*z*	*p*-Value	Effect Size (*r*) ^2^
Total (37 maximum score)	26.0 [24.0, 28.0]	17.0 [15.0, 20.0]	105.5	−7.3	<0.0001	0.77
Servings	3.0 [2.0, 4.0]	2.0 [1.0, 3.0]	602.0	−3.5	0.001	0.36
Food choices	18.0 [16.3, 19.0]	13.0 [11.0, 15.0]	163.0	−6.9	<0.0001	0.72
Portions	3.4 [3.0, 4.0]	2.0 [1.00, 3.00]	643.0	−5.3	<0.0001	0.56
Resources	1.0 [1.0, 2.0]	0.0 [0.0, 1.0]	301.5	−6.2	<0.0001	0.65

^1^ Data given as median (25th, 75th percentile). ^2^ Calculated using Rosenthal’s [43] formula; Cohen’s [44] effect sizes for *r* were small (0.1), medium (0.3), large (0.5). Note. Scoring: correct response: +1; incorrect response: 0; unsure response or “choose not to answer”: 0. Maximum possible scores: total = 37; servings = 6; food choices = 25; portions = 5; resource = 1.

**Table 2:** Median-split table for nutrition and non-nutrition students’ total nutrition knowledge scores (*n* = 91).

	Median or Above (*n*)	Below Median (*n*)
Nutrition students (*n* = 40)	40	0
Non-nutrition students (*n* = 51)	9 [2.0, 4.0]	42 [1.0, 3.0]

**Table 3:** Test-retest reliability using Pearson’s product-moment correlation (*n* = 35; nutrition students only).

Knowledge Section ^1^	Pearson’s Product-Moment Correlation (*r*) ^2^	*p*-Value
Total	0.50	0.002
Servings	0.78	<0.001
Food choices	0.43	0.001
Portions	0.54	0.001

^1^ Only results for medium to large correlations shown. ^2^ Cohen’s 31 small (0.1), medium (0.3) and large (0.5) correlations (*r*) were used.

## Supplementary Materials

The following are available online at https://www.mdpi.com/2072-6643/12/7/1964/s1, Supplementary S1: Questionnaire to assess knowledge related to pre-schoolers’ nutrition.
