# Supplementary material for: The Development of a Psychometrically Valid and Reliable Questionnaire to Assess Nutrition Knowledge Related to Pre-Schoolers
Source: Nutrients. 2020 Jul 1;12(7):1964. doi: 10.3390/nu12071964 (PMC7400463; doi:10.3390/nu12071964)
Supplement: Supplementary file 1 [file nutrients-12-01964-s001.pdf]

INTRODUCTION

<u>Nutrition and physical activity knowledge and perspective questionnaire within ECE</u>

Thank you for taking time to complete this anonymous questionnaire. It should take approximately 15 minutes to complete. The information you provide will be kept confidential.

Please answer all questions <u>yourself</u>. If you do not know the answer, mark "not sure". **Please do not guess.**
This is **<u>not a test.</u>** Your answers will help us identify what is known about nutrition and physical activity for children aged 2-5 years.

If you would like further information about the study and your rights, please click the following link: http://ecenutritionknowledge.wordpress.com

Committee Approval Statement *This project has been reviewed and approved by the Massey University Human Ethics Committee: Northern, Application 15/36. If you have any concerns about the conduct of this research, please contact Mr Jeremy Hubbard, Chair, Massey University Human Ethics Committee: Southern A, telephone 04 801 5799, X 63487, email humanethicsoutha@massey.ac.nz.*

End of Block

SERVINGS

<u>We want to know about SERVINGS of food groups</u>

Note: Serving sizes are provided next to each question
A serving <u>does not necessarily</u> need to be eaten all in one sitting but can be spread throughout the day

---

Examples of 1 serving:

- 1 apple, pear, banana or orange
- 2 small apricots or plums
- ½ cup of fresh fruit salad
- ½ cup of stewed or canned fruit

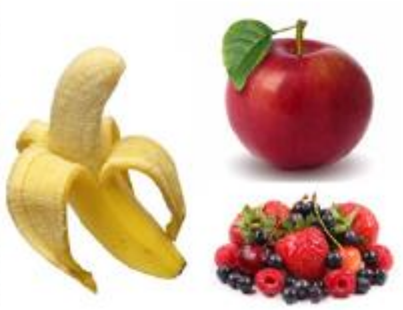

1. How many <u>servings of fruit</u> should a 2-5 year old child eat each day? (Please choose one from the drop-down box)

- ○ None per day (1)
- ○ At least 1 (2)
- ○ At least 2 (3) - *correct*
- ○ At least 3 (4)
- ○ At least 4 (5)
- ○ At least 5 (6)
- ○ At least 6 (7)
- ○ At least 7 (8)
- ○ At least 8 (9)
- ○ At least 9 (10)
- ○ At least 10 (11)
- ○ Not sure (12)

Examples of 1 serving:

- 1 medium potato, taro or kumara
- ½ cup of cooked vegetables
- ½ cup of salad
- 1 tomato or carrot

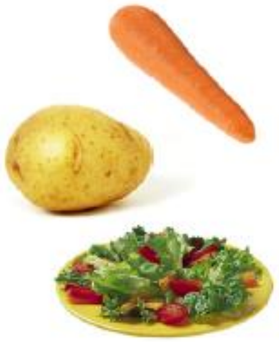

2. How many <u>servings of vegetables</u> should a 2-5 year old child eat each day?
(Please choose one from the drop-down box)

- ○ None per day (1)
- ○ At least 1 (2)
- ○ At least 2 (3) - *correct*
- ○ At least 3 (4)
- ○ At least 4 (5)
- ○ At least 5 (6)
- ○ At least 6 (7)
- ○ At least 7 (8)
- ○ At least 8 (9)
- ○ At least 9 (10)
- ○ At least 10 (11)
- ○ Not sure (12)

What is one serving?

- 1 roll
- 1 medium slice of bread
- 1 cup of cornflakes or rice bubbles or 2 breakfast wheat biscuits
- ½ cup porridge
- 1 cup of cooked pasta, noodles or rice
- 1 cup of cassava or tapioca

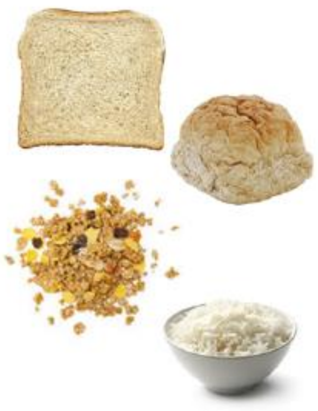

3 (a). How many servings of breads and cereals should a 2-5 year old child eat each day?   (Please choose one from the drop-down box)

- None per day (1)
- At least 1 (2)
- At least 2 (3)
- At least 3 (4)
- At least 4 (5) - *correct*
- At least 5 (6)
- At least 6 (7)
- At least 7 (8)
- At least 8 (9)
- At least 9 (10)
- At least 10 (11)
- Not sure (12)

---

3 (b). How often should a healthy 2-5 year old child eat wholegrain types of breads and cereals?
(Please choose one from the drop-down box)

- Never (1)
- Rarely (2)
- Some days (3)
- Most days (4) - *correct*
- Every day (5)
- Not sure (6)
- Choose not to answer (7)

Examples of 1 serving:

- 2 slices of cooked lean meat, e.g. roast lamb, chicken, beef or pork
- ¾ cup of mince or casserole
- 1 medium fillet of fish or steak
- 2 chicken drumsticks or 1 chicken leg
- 1 medium pāua or kina
- 1 egg
- 3/4 cup baked beans
- ¾ cup of tofu

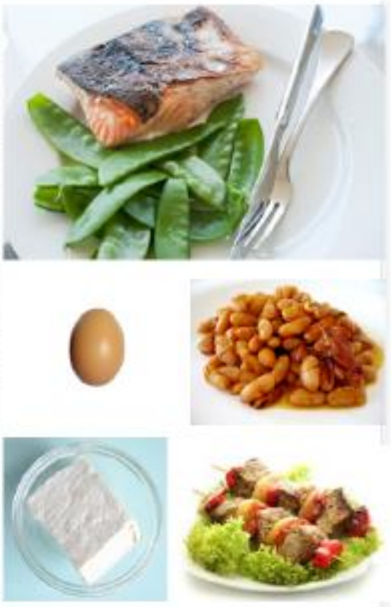

4. How many servings of meats, chicken, seafood, eggs, legumes, nuts and seeds should a 2-5 year old child eat each day? (Please choose one from the drop-down box)

- ○ None per day (1)
- ○ At least 1 (2) *- correct*
- ○ At least 2 (3)
- ○ At least 3 (4)
- ○ At least 4 (5)
- ○ At least 5 (6)
- ○ At least 6 (7)
- ○ At least 7 (8)
- ○ At least 8 (9)
- ○ At least 9 (10)
- ○ At least 10 (11)
- ○ Not sure (12)

What is one serving?

- 1 cup of reduced- or low-fat milk (250ml)
- 1 pottle of reduced- or low-fat yoghurt
- 2 slices or ½ cup of grated cheese, e.g. edam

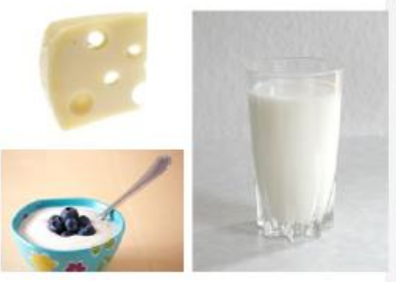

5. How many servings of milk, milk products and calcium-fortified milk alternatives should a 2-5 year old eat each day? (Please choose one from the drop-down box) *Assessor note: if either correct option is selected, give score of +1*

- ○ None per day (1)
- ○ At least 1 (2)
- ○ At least 2 (3) *- correct*
- ○ At least 3 (4) *- correct*
- ○ At least 4 (5)
- ○ At least 5 (6)
- ○ At least 6 (7)
- ○ At least 7 (8)
- ○ At least 8 (9)
- ○ At least 9 (10)
- ○ At least 10 (11)
- ○ Not sure (12)

End of Block

FOOD CHOICES

We want to know about FOOD CHOICES

6 (a). Which of the following beverages are suitable for 2-5 year old children? (Please select one for each) *Assessor note: if either of two possible answers are selected (e.g. never or occasional), score as +1.*

	Never (1)	Occasional (less than once per week) (2)	Sometimes (3)	Everyday (4)	Not sure (5)	Choose not to answer (6)
Water (Q6a_1)				X		
Cow's milk (or alternative) (Q6a_2)				X		
Flavoured milk based drinks (Q6a_3)	X	X				
Fizzy drinks (including diet drinks) (Q6a_4)	X	X				
Cordial or fruit drinks (Q6a_5)	X	X				
Tea or coffee (Q6a_6)	X					
Sports or energy drinks (Q6a_7)	X					

6 (b). Which of the following beverages do you think would be appropriate to give to healthy 2-5 year old child <u>everyday</u> (Please select all that apply): *Assessor note: <u>all</u> appropriate options must be selected to gain a score of +1*

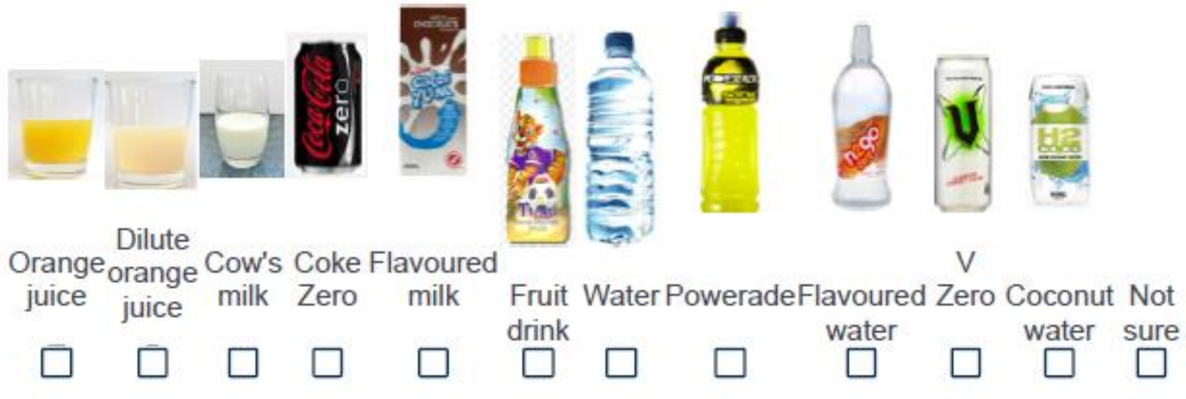

(1) (2) (3) - *correct* (4) (5) (6) (7) - *correct* (8) (9) (10) (11) (12)

---

6 (c). Are there any other beverages that you think are suitable for 2-5 year old children? (Please select one)
Assessor note: an answer of no or yes is considered correct, as along as the items listed align with current nutrition guidelines for pre-schoolers, e.g. Toddler milk, herbal tea and breastmilk

- ○ Yes (please specify): (1) ____
- ○ No (2)
- ○ Choose not to answer (3)

---

7 (a). Which of the following snacks are suitable for 2-5 year old children? (Please select one for each) *Assessor note: if either of two possible answers are selected (e.g. never or occasional), score as +1.*

	Never (1)	Occasional (less than once per week) (2)	Sometimes (3)	Everyday (4)	Not sure (5)	Choose not to answer (6)
Potato crisps, corn snacks or chips (e.g. burger rings, rashuns, etc) (Q7a_1)		X				
Fruit (Q7a_2)				X		
Chocolate coated or cream filled biscuits (Q7a_3)		X				
Biscuits (e.g. plain, semi-sweet, gingernut, shortbread) (Q7a_4)		X	X			
Yoghurt pottle (all types) (Q7a_5)				X		
Vegetable sticks (Q7a_6)				X		
Nuts (all types) (Q7a_7)	X					
Hard dried fruits (e.g. banana chips) (Q7a_8)	X					
Soft dried fruits (e.g. raisins) (Q7a_9)			X			
Ice-cream (Q7a_10)		X				
Cheese (e.g. cheddar, colby, etc) (Q7a_11)				X		
Pastries (e.g. mini meat pie/sausage roll) (Q7a_12)		X				
Plain crackers or crispbreads (Q7a_13)			X			

Q7b 7 (b). Are there any other snacks that you think are suitable for 2-5 year old children? (Please select one)
Assessor note: an answer of no or yes is considered correct, as along as the items listed align with current nutrition guidelines for pre-schoolers, e.g. snacks that are low in fat, sugar and salt + low choking risk.

- ○ Yes (please specify) (1) ____
- ○ No (2)
- ○ Choose not to answer (3)

---

7 (c). How many snacks should a preschooler have per day? *Assessor note: if either correct option is selected, give score of +1*

- ○ None per day (1)
- ○ At least 1 (2)
- ○ At least 2 (3) *- correct*
- ○ At least 3 (4) *- correct*
- ○ At least 4 (5)
- ○ At least 5 (6)
- ○ At least 6 (7)
- ○ More than 6 per day (8)
- ○ Not sure (9)

---

8. Select food items that you think belong in a healthy lunch for a 2-5 year old in an ECE setting (please select all that apply):
Assessor note: <u>all</u> appropriate options must be selected to gain a score of +1

☐ 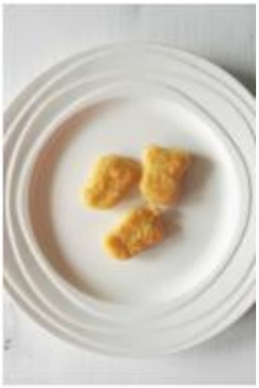 **Fried chicken nuggets** (1)

☐ 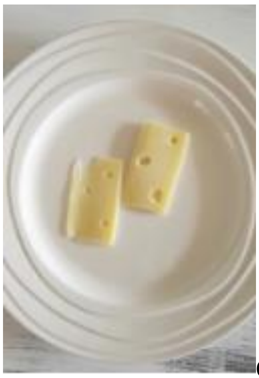 **Cheese** (2) *- correct*

☐ 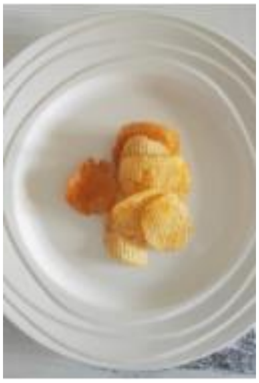 **Potato crisps** (3)

☐ 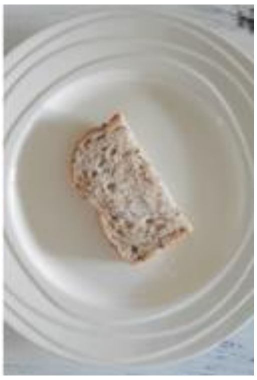 **Wholemeal bread** (4) *- correct*

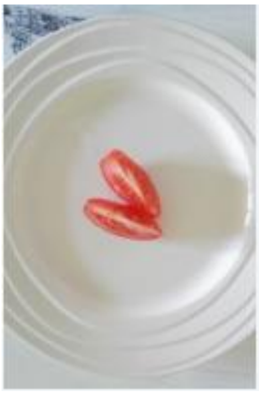

☐ **Tomato** (5) *- correct*

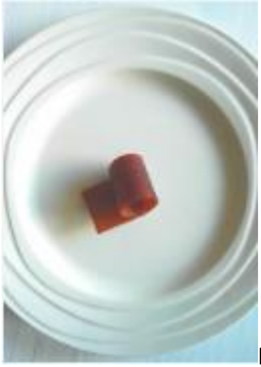

☐ **Fruit roll up** (6)

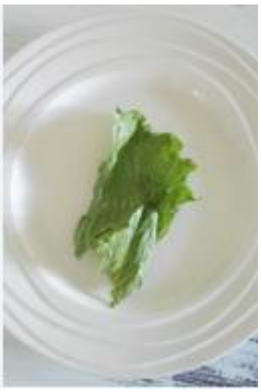

☐ **Lettuce** (7) *- correct*

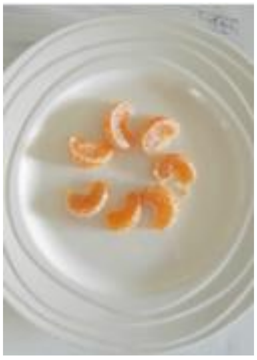

☐ **Mandarin** (8) *- correct*

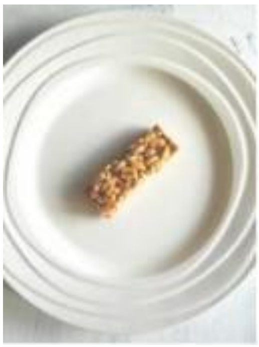

☐ **Fruit & nut muesli bar** (9)

☐ ⊗**Not sure** (10)

9 (a). Which of the following is an example of one recommended serving of dairy for a 2-5 year old child? (Please select one)

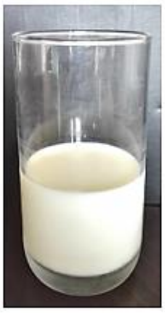

1/2 cup cow's milk (100-120ml)

(1)

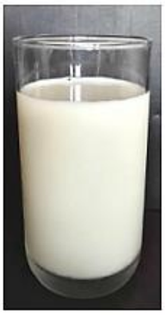

1 cup cow's milk (250ml)

(2) *- correct*

Not sure

(3)

9 (b). Which of the following is an example of one recommended serving of fruit for a 2-5 year old child? (Please select one)

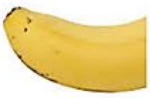

Half banana

(1)

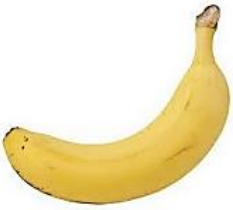

One medium banana

(2) *- correct*

Not sure

(3)

9 (c). Which of the following is an example of one recommended serving of vegetables for a 2-5 year old child? (Please select one)

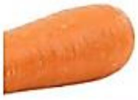

Half carrot

(1)

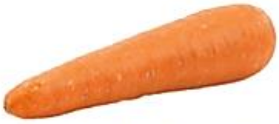

One medium carrot

(2) *- correct*

Not sure

(3)

9 (d). Is the following an example of one portion OR one recommended serving of breads and cereals for a 2-5 year old child? (Please select one)

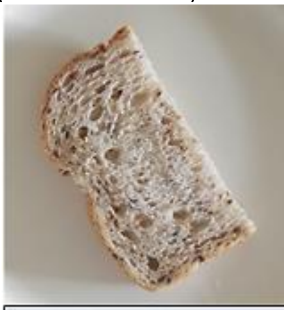

½ slice of bread

- One **portion** (1) *- correct*
- One **serving** (2)
- Not sure (3)

---

9 (e). Is the following an example of one portion OR one recommended serving of meat for a 2-5 year old child? (Please select one)

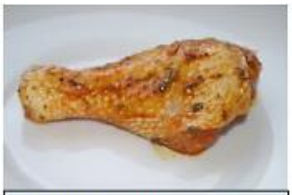

1 chicken drumstick

- One **portion** (1) *- correct*
- One **serving** (2)
- Not sure (3)

End of Block

Nutrition Perspectives

We want to know about YOUR PERSPECTIVES about feeding

10. Please read each statement below and select the response that best describes your perspective

	Strongly Disagree (1)	Disagree (2)	Somewhat disagree (3)	Neither agree nor disagree (4)	Somewhat agree (5)	Agree (6)	Strongly agree (7)	Not sure (8)	Choose not to answer (9)
Mealtimes should be fun (Q10_1)									
Preschoolers should eat together (Q10_2)									
Snacks should be low in sugar (Q10_3)									
Preschoolers should have a choice of a variety of foods at mealtimes (Q10_4)									
Preschoolers should always eat all the food on their plate (Q10_5)									
ECE teachers should role model healthy eating to preschoolers (Q10_6)									
ECE teachers should eat with preschoolers (Q10_7)									
ECE teachers should talk to preschoolers about what they are eating (Q10_8)									

ECE teachers should encourage preschoolers to try new foods (Q10_9)
It is important that preschoolers are involved in an edible garden in ECE settings (Q10_10)
ECE teachers play a vital role in promoting nutrition to preschoolers (Q10_11)
A preschooler's healthy eating is more of the parent's responsibility (Q10_12)
Childcare centres have a role in supporting parents with information and ideas on healthy eating (Q10_13)
The food children eat in my centre provides them with the right fuel they need for the day (Q10_14)
I feel confident having conversations with parents about food and nutrition (Q10_15)

Celebrations and events in our centre should support healthy eating messages (Q10_16)	
Healthy eating education is part of Tē-whariki and can support other areas of learning (Q10_17)	

11. Do we have guidelines from the Ministry of Health for feeding preschoolers? (Please select one)

- Yes (1) *- correct*
- No (2)
- Not sure (3)
- Choose not to answer (4)

Display This Question: (Skip-logic)

If 11. Do we have guidelines from the Ministry of Health for feeding preschoolers? (Please select one) = Yes

12. Have **you** used these guidelines? (Please select one)

- Yes (1)
- No (2)
- Not sure (3)
- Choose not to answer (4)

13. If needed, where would **you** source information about nutrition and feeding practices for 2-5 year olds? (Please select all that apply)

- ☐ Ministry of Health (1)
- ☐ Ministry of Education (2)
- ☐ Family/whānau (3)
- ☐ Friends (4)
- ☐ Heart Foundation NZ (5)
- ☐ Internet/Google search (6)
- ☐ ECE colleagues (7)
- ☐ Plunket nurse (8)
- ☐ Registered Dietitian (9)
- ☐ Doctor (10)
- ☐ Regional Public Health service (11)
- ☐ ⊗Choose not to answer (12)
- ☐ Other (Please specify): (13) ____

---

14. Do any of these factors make it challenging **within ECE services** to promote healthy eating to preschoolers? (Please select all that apply)

- ☐ Lack of staff training (1)
- ☐ Insufficient funds (2)
- ☐ Insufficient time to teach nutrition (3)
- ☐ Lack of support from parents/families/whānau (4)
- ☐ Lack of support from colleagues (5)
- ☐ Lack of resources/information on healthy food for children (6)
- ☐ Sales of unhealthy (high fat, salt or sugar) foods as fundraisers (7)
- ☐ Lack of staff confidence talking about nutrition (8)
- ☐ Concerns about food intolerance or allergies (9)
- ☐ ⊗No, there are no factors that make is challenging within an ECE to promote healthy eating to preschoolers (10)
- ☐ ⊗Choose not to answer (11)
- ☐ Other (please specify): (12) ____

---

15. Are there any factors that make is difficult for **you** to know about healthy eating guidelines for preschoolers? (Please select all that apply)

- ☐ Lack of staff training (1)
- ☐ Insufficient funds (2)
- ☐ Lack of support from parents/families/whānau (3)
- ☐ Lack of support from colleagues (4)
- ☐ Nutrition policies in my centre (5)
- ☐ Lack of resources/information on healthy food for children (6)
- ☐ Lack of confidence talking about nutrition (7)
- ☐ Confusing nutrition messages in the media (8)
- ☐ ⊗No, there are no factors that make it difficult for me to know about healthy eating for preschoolers (9)
- ☐ ⊗Choose not to answer (10)
- ☐ Other (please specify): (11) ____

---

16. Is there anything else that you would like to tell us regarding food, nutrition and feeding practices in ECE?

____

____

End of Block

PHYSICAL ACTIVITY

We want to know about YOUR PERSPECTIVES about physical activity

17. Read each statement below and select the response that best describes your perspectives.

	Strongly Disagree (1)	Disagree (2)	Somewhat disagree (3)	Neither agree nor disagree (4)	Somewhat agree (5)	Agree (6)	Strongly agree (7)	Not sure (8)	Choose not to answer (9)
I am able to define the term "physical activity" (Q17_1)									
I am able to define the term "fundamental movement skills" (Q17_2)									
Physical activity is important for all preschoolers' brain and motor skill development (Q17_3)									
I know what activities will develop specific movement skills (Q17_4)									
I understand the difference between structured and unstructured play (Q17_5)									
ECE teachers play a vital role in promoting preschoolers' physical activity and									

development (Q17_6)
A preschooler's physical activity and development is more of the parent's responsibility (Q17_7)
I have the skills and abilities I need to support children’s physical activity and development (Q17_8)

18. Do we have any New Zealand physical activity guidelines for preschoolers? (Please select one)

- ○ Yes (1) *- correct*
- ○ No (2)
- ○ Not sure (3)
- ○ Choose not to answer (4)

Display This Question (Skip-logic)

If 18. Do we have any New Zealand physical activity guidelines for preschoolers? (Please select one) = Yes

19. Have **you** used these guidelines? (Please select one)

- ○ Yes (1)
- ○ No (2)
- ○ Not sure (3)
- ○ Choose not to answer (4)

20. If needed, where would **you** source information about physical activity for 2-5 year olds? (Please select all that apply)

- ☐ The Ministry of Health (1)
- ☐ The Ministry of Education (2)
- ☐ Sport New Zealand (3)
- ☐ Heart Foundation NZ (4)
- ☐ Family/whānau (5)
- ☐ Friends (6)
- ☐ Internet/Google search (7)
- ☐ ECE colleagues (8)
- ☐ Plunket nurse (9)
- ☐ Registered Dietitian (10)
- ☐ Doctor (11)
- ☐ Regional Public Health service (12)
- ☐ ⊗Choose not to answer (13)
- ☐ Other (please specify): (14) ____

---

21. Do any of these factors make it challenging **within ECE services** to promote physical activity to preschoolers? (Please select all that apply)

- ☐ Lack of staff training (1)
- ☐ Insufficient funds (2)
- ☐ Insufficient time to teach physical activity (3)
- ☐ Lack of staff confidence to teach physical activity (4)
- ☐ Lack of support from parents/families/whānau (5)
- ☐ Lack of support from colleagues (6)
- ☐ Lack of resources/information on physical activity for children (7)
- ☐ Limited opportunity/space for physical education (8)
- ☐ Limited storage (9)
- ☐ Safety concerns of management/staff (10)
- ☐ Safety concerns of parents (11)
- ☐ Insufficient staffing to supervise active play (12)
- ☐ ⊗No, there are no factors that make is challenging within an ECE to promote physical activity to preschoolers (13)
- ☐ ⊗Choose not answer (14)
- ☐ Other (please specify): (15) ____

---

22. Are there any factors that make it challenging for **you** to know about physical activity guidelines for preschoolers? (Please select all that apply)

- ☐ Lack of staff training (1)
- ☐ Insufficient funds (2)
- ☐ Insufficient time to teach physical activity (3)
- ☐ Lack of confidence to teach physical activity (4)
- ☐ Lack of support from parents/families/whānau (5)
- ☐ Lack of support from colleagues (6)
- ☐ Lack of resources/information on physical activity for children (7)
- ☐ Physical activity policies in my centre (8)
- ☐ ⊗No, I do not experience any challenges to knowing about physical activity guidelines for preschoolers (9)
- ☐ ⊗Choose not answer (10)
- ☐ Other (please specify): (11) ____

---

23. Is there anything else that you would like to tell us regarding <u>physical activity</u> in ECE?

____

____

End of Block

Demographics

INFORMATION ABOUT YOU

What is your gender? (Please choose one from the drop-down box)

○ Male (1)
○ Female (2)
○ Other (3)
○ Choose not to answer (4)

Which ethnic group(s) do you belong to? (Please select all that apply)

☐ New Zealand Māori (1)
☐ Cook Island Māori (2)
☐ Fijian (3)
☐ Niuean (4)
☐ Samoan (5)
☐ Tokelauan (6)
☐ Tongan (7)
☐ Other Pacific Island (8)
☐ New Zealand European/Pakeha (9)
☐ Other European (10)
☐ Chinese (11)
☐ Other Asian (12)
☐ Indian (13)
☐ South East Asian (14)
☐ ⊗Choose not to answer (15)
☐ Other (please specify): (16) ____

What is your age in years? (Please enter in the text box below)

____

Which childcare provider are you currently employed by? (Please choose one from the drop-down box)

- ○ Other (4)
- ○ Choose not to answer (5)

Note. The Ministry of Education ECE directory (August 2017) was directly copied into the questionnaire; other and choose not to answer options were available.

If you selected "other" in the previous question, please specify in the text box below:

____

What is your highest qualification? (Please select one)

- ○ None (1)
- ○ School Certificate / NCEA level 1 (2)
- ○ 6th Form Certificate / NCEA level 2 (3)
- ○ Playcentre Qualification (Level 3 or higher) (4)
- ○ 2 year Diploma (e.g. Kindergarten Diploma, PIECCA Diploma etc) (5)
- ○ 3 year Diploma of Teaching (ECE) (6)
- ○ Bachelor of Education (Teaching) (ECE) (7)
- ○ Bachelor degree in another discipline (please specify): (8)

____

- ○ Graduate Diploma of Teaching (ECE) (9)
- ○ Post-graduate level qualifications (e.g. PhD, Masters, Honours, Post-graduate diploma etc) (10)
- ○ Choose not to answer (11)
- ○ Other (please specify): (12) ____

What is your current role? (Please select one)

- ○ Teacher in training (1)
- ○ Qualified teacher (2)
- ○ Non-qualified teacher (3)
- ○ Cook/chef (4)
- ○ Administrator (5)
- ○ Choose not to answer (6)
- ○ Other (please specify): (7) ____

What is the total number of years and months that you have worked in early childhood settings? (e.g. 2 years 3 months) (Please select one from each drop-down box)

- 0 years (1)
- 1 year (2)
- 2 years (3)
- 3 years (4)
- 4 years (5)
- 5 years (6)
- 6 years (7)
- 7 years (8)
- 8 years (9)
- 9 years (10)
- 10 years (11)
- 11 + years (12)

and

- 0 months (1)
- 1 month (2)
- 2 months (3)
- 3 months (4)
- 4 months (5)
- 5 months (6)
- 6 months (7)
- 7 months (8)
- 8 months (9)
- 9 months (10)
- 10 months (11)
- 11 months (12)

By submitting your questionnaire, you are consenting to take part in this research. Please click the arrow below to submit. If you would not like to take part, please exit this page by closing the browser window.

Additional instructions for the administer: No ⊗ means that if participants select this item, all other items will select off. Numbers in brackets beside options indicate scoring system and were used to code data and do not represent weighted scoring. Correct answer (indicated in red) = +1. Incorrect, choose not to answer or not sure answer = "0"
